# Validity and responsiveness to change of the Active Australia Survey according to gender, age, BMI, education, and physical activity level and awareness

**DOI:** 10.1186/s12889-019-6717-1

**Published:** 2019-04-15

**Authors:** Corneel Vandelanotte, Mitch J. Duncan, Rob Stanton, Richard R. Rosenkranz, Cristina M. Caperchione, Amanda L. Rebar, Trevor N. Savage, W. Kerry Mummery, Gregory S. Kolt

**Affiliations:** 1 Physical Activity Research Group, School of Human, Health and Social Sciences, Central Queensland University, Rockhampton, QLD Australia; 2 School of Medicine & Public Health; Priority Research Centre for Physical Activity and Nutrition, Faculty of Health and Medicine, University of Newcastle, Newcastle, NSW Australia; 3 School of Medical and Applied Sciences, Central Queensland University, Rockhampton, QLD Australia; 4 Department of Food, Nutrition, Dietetics and Health, Kansas State University, Manhattan, KS USA; 5 School of Health and Exercise Sciences, University of Technology Sydney, Sydney, NSW Australia; 6 School of Science and Health, Western Sydney University, Sydney, NSW Australia; 7 Faculty of Physical Education and Recreation, University of Alberta, Edmonton, Alberta Canada

**Keywords:** Measurement, Self-report, Accelerometer, Socio-demographics, Surveillance, Subgroups

## Abstract

**Background:**

This study aimed to investigate the validity of the Active Australia Survey across different subgroups and its responsiveness to change, as few previous studies have examined this.

**Methods:**

The Active Australia Survey was validated against the ActiGraph as an objective measure of physical activity. Participants (*n* = 465) wore the ActiGraph for 7 days and subsequently completed the Active Australia Survey. Moderate activity, vigorous activity and total moderate and vigorous physical activity were compared using Spearman rank-order correlations. Changes in physical activity between baseline and 3-month assessments were correlated to examine responsiveness to change. The data were stratified to assess outcomes according to different subgroups (e.g., gender, age, weight, activity levels).

**Results:**

With regards to the validity, a significant correlation of ρ = 0.19 was found for moderate physical activity, ρ = 0.33 for vigorous physical activity and ρ = 0.23 for moderate and vigorous physical activity combined. For vigorous physical activity correlations were higher than 0.3 for most subgroups, whereas they were only higher than 0.3 in those with a healthy weight for the other activity outcomes. With regards to responsiveness to change, a correlation of ρ = 0.32 was found for moderate physical activity, ρ = 0.19 for vigorous physical activity and ρ = 0.35 for moderate and vigorous physical activity combined. For moderate and vigorous activity combined correlations were higher than 0.4 for several subgroups, but never for vigorous physical activity.

**Conclusions:**

Little evidence for the validity of Active Australia Survey was found, although the responsiveness to change was acceptable for several subgroups. Findings from studies using the Active Australia Survey should be interpreted with caution.

**Trial registration:**

World Health Organisation Universal Trial Number: U111–1119-1755. Australian New Zealand Clinical Trials Registry, ACTRN12611000157976. Registration date: 8 March 2011.

## Background

Regular physical activity reduces the risk for developing chronic diseases, yet large proportions of the population are inactive which leads to an increased burden of disease in Australia [1, 2]. As such, robust physical activity measures are important for epidemiology, surveillance and evaluation of interventions. The most used, cost-effective and feasible method of assessing physical activity in large populations is through using self-report questionnaires [3]. Although limitations associated with self-report measurements are well known [4, 5], and the use of accelerometer-based physical activity monitoring is becoming increasingly feasible [6], self-reported measurement still represents an efficient way to collect data on physical activity in population health research.

The accurate collection of physical activity using self-reported methods is not easy, as it depends on a number of factors. Accuracy relies on participants’ ability to correctly recall physical activity performed in the past, whether participants’ interpretation of physical activity intensity aligns with established definitions for activity intensity, as well as whether survey questions are able to capture these interpretations of intensity [7]. For example, women or older adults may perceive that specific activities of moderate intensity require greater effort than what men and younger adults perceive of the same activities, and therefore rather classify them as being of vigorous intensity [8, 9]. Furthermore, physical activity questionnaires validated for use in one population are often used in different populations or settings in which they have not been validated. It is therefore important to investigate the extent to which the validity of a self-report instrument varies across different populations [5]. If validity differs by population group, then this has important implications for physical activity surveillance. Few studies have examined the accuracy of self-report questionnaires according to socio-demographic factors. While some studies demonstrate that self-reports can be accurate for women and older age adults [9, 10], they did not simultaneously assess validity in men and young adults. Nevertheless, some studies have compared groups and indicated that self-report accuracy decreases when BMI increases and when activity levels increase [7, 11]. More research is however needed to confirm these observations.

During the past 15 years, the Active Australia Survey [12] has been widely used to measure physical activity in Australian and international surveillance studies and large cohort studies [13–16]. The Active Australia Survey assesses frequency (sessions) and duration (minutes) of physical activity in the past week [12]. While correlation coefficients of 0.3 have often been reported as demonstrating acceptable evidence of validity in physical activity research [17–22], a systematic review on the validity of physical activity questionnaires by Helmerhortst et al. (2012) categorised validity as poor when correlations were below 0.4 [23]. The measurement properties of the Active Australia Survey have been assessed, and with correlation coefficients for total physical activity ranging from 0.42 to 0.61 [10, 11], they have been deemed acceptable [9, 10]. Few studies, however, have examined how the validity differs across different subgroups [7, 11, 23]. Furthermore, although the Active Australia Survey was developed for physical activity surveillance [12]; it is nevertheless often used in intervention research with small study groups and detecting change in small groups requires greater measurement sensitivity [24]. Two studies have examined responsiveness to change using the Active Australia survey [25, 26], but only one of these studies examined this in comparison to an objective measure of physical activity [26]. This study found acceptable but lower responsiveness of the Active Australia Survey compared to using accelerometry [26]. Therefore the aims of this study were: 1) to investigate the validity of the Active Australia Survey in different population subgroups from a sample of Australian adults who participated in a randomised controlled trial; and 2) to investigate the responsiveness to change of the Active Australia Survey relative to objective accelerometer assessments.

## Methods

The Edinburgh Validity and Reliability Framework was used to specify what types of validity our study assessed [27]. Specifically, when we refer to ‘validity’ we mean ‘criterion validity’ and when we refer to ‘responsiveness to change’ we mean ‘behavioural reliability’ (i.e., assessment of stability accounting for behavioural changes).

### Participants

All participants in this study were part of the Walk 2.0 trial [28, 29], a three-group randomised controlled trial that assessed the effectiveness of a traditional physical activity promotion website (www.10000steps.org.au), a social networked physical activity promotion website (www.walk.org.au), and a print-based control group. Details of the study methods and procedures of Walk 2.0 have been published previously [28]. A total of 504 participants were recruited via random selection from Australian electoral roll, local print media, and email lists. Eligible participants were inactive English speaking adults (+ 18 years) with Internet access who lived in Western Sydney or Rockhampton. All participants in the Walk 2.0 trial who were randomised into a group were included in this study. A single item physical activity measure was used to screen participant activity levels prior to randomisation [30]. While the aim of the Walk 2.0 study was to only recruit inactive participants, 42.9% met the physical activity recommendations at baseline [29]. We have reported more details on the screening procedure and its limitations elsewhere [31]. The issues with recruiting an inactive sample suggest that many of those recruited were motivated to become more active, and as such they may have been different from the Australian population at large.

### Procedures

Eligible participants were invited to the university, fitted with an ActiGraph activity monitor and instructed to wear it for 7 days. Participants were asked to record wear time and reasons for removing the ActiGraph during the day (e.g. water sports) using a paper-based log. Eight days later participants returned to the university and completed the Active Australia Survey. Before completing the Active Australia Survey, ActiGraph data were inspected; if individual data were invalid, participants were asked to wear the ActiGraph again, until valid data were obtained. The Walk 2.0 trial measured participants using this protocol at 4 time points (0, 3, 12 and 18 months), however the present paper only reports outcomes for baseline (validity) and 3-month (sensitivity to change) time points. Only baseline data were used to assess validity, as the subsequent intervention would have intentionally influenced physical activity at later time points. Only baseline and 3-month time points were used to assess responsiveness to change, as actual physical activity change due to the intervention will have been the greatest immediately after completing the intervention and also because drop-out increased over subsequent time points which may introduce selection bias.

### Measures

The Active Australia Survey: this survey comprises eight items to assess the frequency (number of sessions) and duration (minutes per week) of walking, moderate and vigorous leisure physical activities and vigorous gardening (in at least 10-min bouts) over the preceding 7 days. Acceptable 5-day test-retest reliability has been reported for the Active Australia Survey with reliability coefficients (spearman’s ρ) ranging between 0.43 and 0.80 and agreement scores (Kappa statistics) ranging between 0.40 and 0.83 [11]. Consistent with Active Australia Survey data treatment recommendations, when participants reported spending time in vigorous gardening, these data were not included in any calculations of total and vigorous physical activity [12]. Duration (minutes per week) for walking, moderate and vigorous physical activities were truncated at 840 min [12]. Total minutes for moderate physical activity (which includes walking minutes), vigorous physical activity and total moderate and vigorous physical activity were calculated. Total minutes for moderate physical activity and walking were combined, as several studies have demonstrated that healthy adults’ self-selected walking speed usually corresponds with moderate intensity physical activity [32, 33].

The ActiGraph activity monitor: The ActiGraph (model GT3x; ActiGraph LLC., Florida) was used to objectively measure physical activity. Although accelerometers do not provide a gold-standard measure, they are not subject to the same sources of error as self-reporting, and are well-accepted for providing evidence for the validity of self-report measures [9, 34, 35]. The validity and reliability of the ActiGraph accelerometer has previously been demonstrated in laboratory testing, and compared to other commercially available activity monitors [36, 37]. For example, ActiGraph counts per minute were highly correlated with oxygen uptake (VO_2_) during treadmill running at various speeds (*r* = .88) [38]. During the induction session, participants were instructed on how to wear the ActiGraph, which was worn over their right hip and fastened using an elastic belt.

Demographics: Age, gender and education level were assessed as part of the survey measure, however height and weight were measured by project staff when participants visited the university using Seca 700 balance scales and a Seca 220 measuring rod (Seca GmbH, Hamburg). Participants removed shoes and heavy personal items. The average of 3 consecutive measurements was recorded. Using BMI (kg/m^2^) participants were classified as healthy weight (BMI = 18.5–24.9), overweight (BMI = 25.0–29.9) or obese (BMI ≥ 30) [39]. Educational level was initially assessed in 6 categories, but was collapsed into 3 categories for this study (school education, vocational and technical education, higher education). Educational level may influence the validity and responsiveness of survey instruments. A higher education level may result in better knowledge about physical activity, a better understanding of being active at different intensities and having a better recall of past activities [40]. This is why we examined validity and responsiveness to change according to educational level.

Physical Activity Awareness: Awareness was assessed using the five items that accompany the assessment of the Active Australia Survey [12]. When participants answered 4 or more questions correctly they were categorised as having ‘high physical activity awareness’; if fewer than 4 questions were answered correctly participants were categorised as having ‘Low physical activity awareness’. (In) accurate awareness of one’s own physical activity level (e.g., in relation to meeting physical activity recommendations) may result in socially desirable responses to the self-report physical activity surveys, and this may undermine the validity of the measure [41].

Overlap: The time frame of the Active Australia Survey (last 7 days) overlapped as much as possible with the time when participants were wearing the ActiGraph; however, it was not possible to always have a perfect overlap. As such, ‘optimal overlap’ was considered when there was no more than 2 days of difference between the last day of ActiGraph monitoring and the time the Active Australia Survey was completed (thus 0-, 1- or 2-day gap). ‘Sub-optimal overlap’ was considered when there was a gap of 3 or more days between ActiGraph and Active Australia administration.

### Data reduction

The ActiGraph data were reduced with custom software (a Microsoft Excel macro) that examined each recorded epoch and determined the intensity of physical activity using the number of ActiGraph counts recorded during that epoch. Valid ActiGraph wear time was determined as at least 600 min wear time per day (during waking hours) on a minimum of 5 of the 7 recorded days [28, 29]. Triaxial data were collected in 1-s epochs and aggregated to 60 s. Using the Freedson et al. cut points [42], between 1953 and 5724 counts per minute was classified as moderate physical activity and 5725 or more counts was classified as vigorous physical activity. As such, the total minutes of moderate physical activity, vigorous physical activity, and total moderate and vigorous physical activity were calculated for each day with valid ActiGraph data. The ActiGraph data were checked for outliers, though none where identified. More detailed information about data reduction processes can be found in the study protocol paper [28]. Total moderate and vigorous physical activity was dichotomised in alignment of achieving the minimum recommendation of 150 min of physical activity per week [43]. This dichotomized variable was used to stratify participants into two subgroups; however, the continuous variables described above were used for the correlational analyses.

### Analyses

Descriptive statistics (χ^2^ and *t*-tests) were used to compare participants with valid ActiGraph data to those without valid ActiGraph data for baseline demographics, as well to compare baseline characteristics for participants who had remained in the study at the 3-month time point and those that had dropped out. McNemar tests were conducted to assess whether there were significant differences in the proportion of people classified as meeting or not meeting physical activity guidelines for the 2 separate measures (Active Australia Survey and Actigraph accelerometer). To assess validity, Spearman rank-order correlation coefficients were computed to assess the relationship between the Active Australia Survey outcomes with the ActiGraph outcomes for participants with complete data for both measures (i.e., complete cases analysis). Spearman correlation coefficients were chosen because self-reported physical activity data were not normally or linearly distributed, however the monotonicity assumption was not violated. The use of Spearman correlations is well accepted and common for assessing the validity of physical activity surveys, including the Active Australia Survey, and is valuable when comparing the results to other studies [7, 9–11, 34, 35]. All reported correlations are between corresponding physical activity categories (e.g., ActiGraph vigorous physical activity was correlated with Active Australia vigorous physical activity). To assess responsiveness to change over time, a linear regression model was used to regress the 3-month Active Australia Survey outcomes onto the baseline Active Australia Survey outcomes for each of the 3 variables (moderate, vigorous and moderate + vigorous physical activity); in doing so, the individual residual scores were calculated for each participant. The same procedure was repeated for the 3-month and baseline ActiGraph variables. Finally, Spearman rank-order correlation coefficients were computed between the individual residual scores from the Active Australia Survey and those from the ActiGraph for the 3 variables. This procedure allows reducing measurement error to a larger extent when compared to directly correlating change scores [44, 45]. Due to the large sample size it was possible to stratify the outcomes and assess whether the correlation coefficients differed for several outcomes (e.g. age). Fisher *r* to *Z* transformations (*z*) were applied to assess whether there were significant differences in the correlations between the subgroups [46]. Statistical significance was set at an alpha level of 0.05.

## Results

A total of 504 participants were randomized into the study and 465 had valid ActiGraph data (a minimum of 600 min of wear time on 5 out of 7 days) at the baseline of the RCT. There were no significant differences between participants with or without valid ActiGraph data for all baseline demographics, with the exception of educational attainment: more participants with a higher education had no valid data (χ^2^ = 7.22, *p* = 0.02). There were no significant differences between participants who remained into the study at 3 months and those who had dropped out for all baseline demographics, with the exception of age: more participants with a younger age had dropped out at 3 months (*t* = 3.21; *p* = 0.001). As Table 1 shows, nearly two thirds of participants were female (65.1%), and about three quarters of the sample was either overweight (35.9%) or obese (39.7%). Participants of different educational levels and ages were well represented, though a high number of participants were aged between 50 and 64 years (39.7%). The majority of the sample reported high physical activity awareness (63.5%); and optimal overlap between the ActiGraph measurement and the Active Australia Survey measurement was achieved in 55.1%. At baseline, similar proportions of participants engaged in 150 min of moderate to vigorous physical activity according to the Active Australia Survey (43.8%) and ActiGraph (44.7%); these proportions were not significantly different (McNemar test = 0.00; *p* = 1.00). However, at 3 months there was a larger gap between the two assessments (61% for Active Australia Survey and 52.7% for ActiGraph), and these differences were significantly different (McNemar test = 8.37; *p* = 0.004). The increase in moderate to vigorous physical activity from baseline to 3 months was 87 min per week according to the Active Australia Survey and 28 min per week according to the Actigraph; the difference in change over time between the two measures was significant (*t* = 3.16; *p* = 0.002).

**Table 1:** Participant demographic characteristics and physical activity levels

N (%)	Total group	Men	Women
Male	176 (34.9)	–	–
Female	328 (65.1)	–	–
18 to 49 years of age	162 (32.1)	41 (23.3)	121 (36.9)
50 to 64 years of age	200 (39.7)	71 (40.3)	129 (39.3)
Over 65 years of age	142 (28.1)	64 (36.4)	78 (23.8)
School education	140 (27.8)	39 (22.2)	101 (30.8)
Vocational and technical education	193 (38.3)	83 (47.2)	110 (33.5)
Higher education	171 (33.9)	54 (30.7)	117 (35.7)
Healthy weight	123 (24.4)	31 (17.6)	92 (28.0)
Overweight	181 (35.9)	76 (43.2)	105 (32.0)
Obese	200 (39.7)	69 (39.2)	131 (39.9)
High physical activity awareness	320 (63.5)	102 (58.0)	218 (66.5)
Low physical activity awareness	184 (36.5)	74 (42.0)	110 (33.5)
Optimal overlap AAS & ActiGraph	256 (55.1)	90 (55.6)	166 (54.8)
Sub-optimal overlap AAS & ActiGraph	209 (44.9)	72 (44.4)	137 (45.2)
Active Australia Survey:			
= < 150 min MVPA week – baseline	283 (56.2)	93 (52.8)	190 (57.9)
> 150 min MVPA week – baseline	221 (43.8)	83 (47.2)	138 (42.1)
= < 150 min MVPA week – 3 months	153 (39.0)	49 (34.0)	104 (41.9)
> 150 min MVPA week – 3 months	239 (61.0)	95 (66.0)	144 (58.1)
ActiGraph Accelerometer:			
= < 150 min MVPA week – baseline	257 (55.3)	72 (44.4)	185 (61.1)
> 150 min MVPA week – baseline	208 (44.7)	90 (55.6)	118 (38.9)
= < 150 min MVPA week – 3 months	177 (47.3)	43 (31.6)	134 (56.3)
> 150 min MVPA week – 3 months	197 (52.7)	93 (68.4)	104 (43.7)
Mean ± SD	Total group (*N* = 504)	Men (*n* = 176)	Women (*n* = 328)
Baseline Active Australia Survey			
Moderate PA (min/week)	163 ± 190	187 ± 218	150 ± 172
Vigorous PA (min/week)	46 ± 93	65 ± 122	36 ± 72
MVPA (min/week)	210 ± 243	253 ± 286	186 ± 213
Three month Active Australia Survey			
Moderate PA (min/week)	238 ± 238	284 ± 281	211 ± 205
Vigorous PA (min/week)	59 ± 110	73 ± 136	51 ± 91
MVPA (min/week)	297 ± 296	358 ± 369	262 ± 238
Baseline ActiGraph			
Moderate PA (min/week)	163 ± 122	190 ± 119	148 ± 122
Vigorous PA (min/week)	4.4 ± 19.6	4.6 ± 19.2	4.4 ± 19.9
MVPA (min/week)	167 ± 127	195 ± 124	153 ± 127
Three month ActiGraph			
Moderate PA (min/week)	187 ± 140	226 ± 152	164 ± 127
Vigorous PA (min/week)	8.1 ± 40.2	9.8 ± 49.5	7.3 ± 33.8
MVPA (min/week)	195 ± 151	236 ± 167	171 ± 136

Note: *AAS* Active Australia Survey, *MVPA* Moderate + Vigorous Physical Activity, *PA* Physical Activity, *SD* Standard Deviation

While nearly all correlation coefficients assessing the validity between the Active Australia Survey and the ActiGraph were significant, they were generally small (see Table 2). For the total group, a correlation of ρ = 0.19 (*p* = 0.000; CI 95% = 0.13–0.32) was found for moderate physical activity, ρ = 0.33 (p = 0.000; CI 95% = 0.11–0.29) for vigorous physical activity and ρ = 0.23 (p = 0.000; CI 95% = 0.24–0.45) for moderate and vigorous physical activity combined. This general pattern, whereby the correlations for vigorous activity were higher than for the other physical activity categories, was relatively similar when the data were stratified according to different subgroups (see Table 2). Few significant differences between subgroups were observed. There were significant differences in the correlations for vigorous physical activity between men and women (*z* = 2.01; *p* = 0.04), as well as between participants aged from 50 to 64 years and those aged over 65 (*z* = 2.04; *p* = 0.04). There was a significant difference in the correlation for moderate intensity physical activity between those of a healthy weight and those who were overweight (*z* = 2.04; *p* = 0.04).

**Table 2:** Spearman Rank Correlations between baseline measures for the Active Australia Survey and the ActiGraph Accelerometer

	Moderate PA	Vigorous PA	MVPA
	ρ (*p*)	ρ (*p*)	ρ (*p*)
	(95% CI)	(95% CI)	(95% CI)
Total group (N = 465)	.199 (000)***	.331 (.000)***	.231 (.000)***
	(.106–.290)	(.241–.415)	(.132–.320)
Men (*n* = 162)	.153 (.053)	.218 (.005)**	.169 (.031)*
	(−.012–.315)	(.069–.367)	(.013–.326)
Women (*n* = 303)	.203 (.000)***	.396 (.000)***	.236 (.000)***
	(.079–.313)	(.288–.504)	(.110–.348)
18 to 49 years of age (*n* = 147)	.228 (.006)**	.363 (.000)***	.263 (.001)**
	(.054–.391)	(.202–.519)	(.074–.426)
50 to 64 years of age (*n* = 186)	.235 (.001)**	.412 (.000)***	.281 (.000)***
	(.102–.384)	(.274–.537)	(.146–.429)
Over 65 years of age (*n* = 132)	.198 (.023)*	.201 (.021)*	.214 (.014)*
	(.024–.366)	(.016–.380)	(.048–.375)
School education (*n* = 135)	.297 (.000)***	.286 (.001)**	.287 (.001)**
	(.119–.460)	(.116–.444)	(.111–.450)
Vocational and technical education (*n* = 179)	.203 (.006)**	.312 (.000)***	.216 (.004)**
	(.050–.360)	(.176–.448)	(.056–.364)
Higher education (*n* = 151)	.084 (.302)	.393 (.000)***	.191 (.019)*
	(−.090–.256)	(.230–.537)	(.027–.355)
Healthy weight (*n* = 114)	.344 (.000)***	.307 (.001)**	.361 (.000)***
	(.164–.515)	(.134–.475)	(.181–.528)
Overweight (*n* = 168)	.108 (.165)	.286 (.000)***	.165 (.032)*
	(−.059–.274)	(.151–.429)	(−.003–.328)
Obese (*n* = 183)	.183 (.013)*	.384 (.000)***	.192 (.000)***
	(.046–.312)	(.240–.523)	(.052–.324)
High physical activity awareness (*n* = 297)	.205 (.000)***	.288 (.000)***	.217 (.000)***
	(.092–.304)	(.170–.397)	(.103–.321)
Low physical activity awareness (n = 168)	.181 (.019)*	.377 (.000)***	.234 (.002)**
	(.021–.340)	(.230–.509)	(.087–.393)
Optimal overlap AAS & ActiGraph (*n* = 256)	.208 (.001)**	.394 (.000)***	.256 (.000)***
	(.084–.331)	(.266–.502)	(.140–.375)
Sub-optimal overlap AAS & ActiGraph (*n* = 209)	.182 (.008)**	.258 (.000)***	.196 (.004)**
	(.044–.316)	(.119–.393)	(.054–.332)
= < 150 min MVPA week ActiGraph (*n* = 257)	.144 (.021)*	.379 (.000)***	.165 (.008)**
	(.017–.262)	(.258–.481)	(.042–.284)
> 150 min MVPA week ActiGraph (*n* = 208)	.228 (.001)** (.096–.355)	.283 (.000)*** (.142–.416)	.247 (.000)*** (.106–.370)

Note: *p* < 0.05 = *; *p* < 0.01 = **; *p* < 0.001 = ***

*AAS* Active Australia Survey, *MVPA* Moderate + Vigorous Physical Activity, *PA* Physical Activity

Compared to the validity correlations, the correlations expressing responsiveness to change were somewhat higher, though still relatively small (see Table 3). For the total group, a correlation of ρ = 0.35 (*p* = 0.000; CI 95% = 0.25–0.45) was found for moderate physical activity, ρ = 0.32 (*p* = 0.001; CI 95% = 0.22–0.43) for vigorous physical activity and ρ = 0.19 (*p* = 0.000; CI 95% = 0.07–0.30) for moderate and vigorous physical activity combined. This general pattern, whereby the correlations for vigorous activity were lower than for the other physical activity categories, was relatively similar when the data were stratified according to different subgroups (see Table 3). No significant differences between subgroups were observed.

**Table 3:** Spearman Rank Correlations of residual scores expressing change between baseline and 3 months

	Moderate	Vigorous	MVPA
	ρ (*p*)	ρ (*p*)	ρ (*p*)
	(95% CI)	(95% CI)	(95% CI)
Total group (*n* = 333)	.325 (.000)***	.189 (.001)**	.356 (.000)***
	(.221–.431)	(.074–.304)	(.253–.454)
Men (*n* = 123)	.413 (.000)***	.213 (.018)*	.435 (.000)***
	(.247–.558)	(.019–.396)	(.262–.573)
Women (*n* = 210)	.251 (.000)***	.174 (.012)*	.298 (.000)***
	(.119–.383)	(.037–.325)	(.160–.433)
18 to 49 years of age (*n* = 95)	.286 (.005)**	.195 (.058)	.366 (.000)***
	(.095–.462)	(−.034–.405)	(.178–.526)
50 to 64 years of age (*n* = 134)	.355 (.000)***	.123 (.156)	.359 (.000)***
	(.194–.506)	(−.069–.303)	(.196–.509)
Over 65 years of age (*n* = 104)	.323 (.001)**	.308 (.001)**	.336 (.000)***
	(.120–.495)	(.128–.474)	(.130–.514)
School education (*n* = 90)	.284 (.007)**	.220 (.038)*	.282 (.007)**
	(.085–.477)	(.003–.420)	(.077–.476)
Vocational and technical education (*n* = 132)	.387 (.000)***	.230 (.008)**	.457 (.000)***
	(.236–.522)	(.031–.409)	(.320–.579)
Higher education (*n* = 111)	.307 (.001)**	.133 (.165)	.343 (.000)***
	(.113–.484)	(−.074–.347)	(.150–.518)
Normal weight (*n* = 78)	.421 (.000)***	.258 (.023)*	.477 (.000)***
	(.200–.599)	(.036–.470)	(.288–.658)
Overweight (*n* = 128)	.217 (.014)*	.244 (.005)**	.278 (.001)**
	(.036–.379)	(.056–.418)	(.098–.445)
Obese (*n* = 127)	.362 (.000)***	.070 (.435)	.346 (.000)***
	(.183–.524)	(−.142–.272)	(.149–.515)
High physical activity awareness (*n* = 217)	.324 (.000)***	.160 (.018)*	.361 (.000)***
	(.194–.450)	(.008–.309)	(.234–.484)
Low physical activity awareness (*n* = 116)	.310 (.001)**	.221 (.017)*	.317 (.001)**
	(.134–.474)	(.014–.400)	(.130–.489)
= < 150 min MVPA week ActiGraph (*n* = 183)	.279 (.000)***	.172 (.020)*	.321 (.000)***
	(.128–.433)	(.007–.343)	(.173–.473)
> 150 min MVPA week ActiGraph (*n* = 150)	.382 (.000)***	.237 (.004)**	.415 (.000)***
	(.230–.522)	(.079–.393)	(.261–.554)

Note: *p* < 0.05 = *; *p* < 0.01 = **; *p* < 0.001 = ***

*MVPA* Moderate + Vigorous Physical Activity, *PA* Physical Activity

## Discussion

The aim of this study was to investigate the validity of the Active Australia Survey stratified for different population subgroups, and to examine its responsiveness to change over time. Overall, the results of this study provide little evidence for the validity of the Active Australia Survey. The correlation coefficients in this study are lower than 0.4, which is considered as poor by Helmerhorst et al. (2012) [23]. Furthermore, they are, for most variables, also lower than 0.3, which has been reported as the lower limit for demonstrating acceptable evidence of validity for self-report physical activity measures [17]. The present results are in contrast to most other Active Australia Survey validation studies using accelerometers, as they reported correlation coefficients for total physical activity ranging from 0.42 to 0.61 [10, 11]. Only 2 studies conducted by Timperio et al. reported correlations below 0.3 [7, 47]. One possible explanation for the contrasting findings could be that the Active Australia Survey was administered differently across studies (e.g., telephone vs. paper-and-pencil administration), however previous studies have found similar correlations irrespective of the administration method [10, 11]. It is noteworthy to point out that all the studies that found acceptable validity levels had smaller samples (range: 44–76), whereas the present study (*n* = 465) and those of Timperio (*n* = 122 and 191) had considerably more participants [7, 47]. When comparing the validity to other physical activity questionnaires, the outcomes of the present study are in line with those of the systematic review of Helmerhorst et al. [23]; median Spearman correlation coefficients for surveys assessed in adults ranged from 0.27 to 0.30 for ‘older’ and ‘newer’ physical activity surveys respectively. Those authors concluded that it appears almost impossible to obtain a valid estimation of a highly variable behavior such as physical activity by self-report [23].

The present study found somewhat higher correlation coefficients in women compared to men (only significant for vigorous physical activity); and while two previous studies demonstrated acceptable validity in women using the Active Australia Survey, they did not compare these outcomes with men [10, 34]. However, the study by Timperio et al. found lower correlations for women compared to men [7]. These differences may be due to gender-based differences in the perception of intensity or gender-based differences in recall or attention to detail [48]. The present study found the lowest correlations between the two measures for those with the highest age (only significant for vigorous physical activity). This is in contrast to a study that found acceptable validity (*ρ* = 0.42) in participants over the age of 65 [9]. Unfortunately their study did not include younger age groups. Cognitive degeneration has been suggested as a reason why accurate physical activity recall may decline in old age [23]. Alternatively, the lower correlations in older participants may be due to changes in the perception of physical activity intensity, whereby activities of moderate intensity may be perceived as vigorous by some, but not by others. No other studies have compared correlations for those with different education levels, and the outcomes of this study suggest that having a higher education does not necessarily reflect better behavioral recall, as correlations were often higher for those with a lower education; moreover, the differences between all age groups were not significant. Counterintuitive outcomes were found for the level of physical activity awareness, as lower physical activity awareness often resulted in higher validity scores (though these differences were not significant). Perhaps a lack of awareness results in lower social desirability bias. With the exception of vigorous physical activity in obese participants, the correlations were lower for those with higher weight (the differences were significant for moderate intensity physical activity). The study by Timperio also examined validity levels according to weight status [7], and found a high level of variability across multiple categories that do not align with the variables of the present study, making between-study comparisons difficult. Fjedlsoe et al. indicated that the validity of the Active Australia Survey decreases when participants are more active [11]. The findings of our study are in line with those of Fjedlsoe et al., but only for vigorous physical activity and the differences were not significant [11]. Fjeldsoe et al. indicate that a widening in measurement error and bias may be responsible for the lower validity in highly active participants [11]. Finally, it is not surprising to find somewhat higher correlations when both measures cover the same measurement period, though the differences were small, not significant and almost negligible when compared to the correlations of the total group.

In broad-reaching physical activity interventions, where modest (but clinically meaningful) changes in behavior are often observed, the responsiveness of self-report measures to detect such changes is critical [25]. The correlations expressing responsiveness to change over time were generally low, although they were somewhat higher than the validation correlations and, as Table 3 shows, for some categories they were higher than 0.4, which indicates a degree of acceptability [23]. For example, correlations higher than 0.4 were observed in men, healthy weight participants, those with vocational or technical education, and those who engage in more than 150 min of moderate and vigorous physical activity according to the ActiGraph for moderate to vigorous physical activity. To our knowledge, only two studies have attempted to examine the responsiveness to change for the Active Australia Survey. Reeves et al. found good responsiveness to change for moderate to vigorous physical activity relative to a more detailed self-report measure (CHAMPS) [25]. In their study (*n* = 381) the responsiveness index (based on Tuley’s formulae) of the Active Australia Survey was 0.50 (95%CI: 0.30–0.69) which was considered as good responsiveness. Lee et al. used the same methodology (i.e., responsiveness index based on Tuley’s formulae) and found a similar responsiveness for the Active Australia Survey (0.45; 95%CI: 0.26–0.65), although it was somewhat lower than the responsiveness for the Actigraph in the same study (0.49; 95%CI: 0.23–0.74) [26]. Given the scarcity of studies assessing responsiveness to change, however, further research is required to confirm these findings.

The large study sample, which allowed stratifying the outcomes for specific subgroup populations, examining responsiveness to change, and the robust study protocol were strengths of this study. However, those who participated in this study were part of a convenience sample recruited to participate in a randomized controlled trial. This may have introduced bias, limiting generalisability of the findings. It should be pointed out, however, that the study sample was well balanced in terms of gender, age, education and weight status. Caperchione et al. provide an in-depth description of the sample of this study and how it compares with the general Australian population [49]. Correlation coefficients can be affected when floor or ceiling effects are present (when more than 15% of the sample reports the highest or lowest possible score) [50]. As such, it is a limitation that floor effects were observed for the vigorous physical activity variables. However, no other floor or ceiling effects were observed for any other variables. Another limitation is that ‘optimal overlap’ for the Active Australia Survey and the ActiGraph measurement was not achieved for all participants. Other Active Australia Validation studies have also reported to this problem [10, 34]. As discussed earlier, this only had a small influence on the observed correlations. To make sure, however, we did run the analyses stratified for all the specific population subgroups with only those participants demonstrating ‘good overlap’. The differences in correlations with the currently presented outcomes were minimal, not warranting the large drop in sample size, ensuring each cell had a large number of participants. Finally, while the ActiGraph is acceptable and often used to assess validity of self-report measures, it is not a gold standard and not able to measure all types of physical activity accurately, this may have reduced the observed correlations [24]. Furthermore, the error associated with regression equations used to derive cut-points for moderate and vigorous-intensity physical activity is also limitation of using accelerometers [4, 6].

## Conclusions

This study provided little evidence for the validity of the Active Australia Survey, although the responsiveness to change was marginally better and deemed acceptable for a number of specific subgroups. The findings are largely in contrast to other Active Australia Survey validation studies with smaller study samples; however they are in line with studies with larger samples sizes, and a review that assessed a range of different physical activity measures. Despite its practicality and low cost, findings from studies that use the Active Australia Survey should always be interpreted with a degree of caution.

## Abbreviation

BMI: Body Mass Index
